# Pulmonary adenocarcinoma presenting with penile metastasis: a case report

**DOI:** 10.1186/1752-1947-6-252

**Published:** 2012-08-21

**Authors:** Christos Karanikas, Nikolaos Ptohis, Evgenia Mainta, Christos S Baltas, Dimitris Athanasiadis, Simos Lechareas, Nikolaos Katirtzoglou, Spyros Xynogalos

**Affiliations:** 1 Radiology Imaging Department, “G. Gennimatas” General Hospital, 154 Mesogeion Avenue, Athens, 11527, Greece; 2 Oncology Department, “G. Gennimatas” General Hospital, 154 Mesogeion Avenue, Athens, 11527, Greece

**Keywords:** Penis, Metastasis, Lung, Adenocarcinoma

## Abstract

**Introduction:**

Penile metastases are an extremely rare occurrence, and most primary malignancies are located in the urinary bladder, prostate, rectum, and rectosigmoid. Although very few cases of penile metastases have been reported, those of lung cancer as the primary tumor are very rare. Among the latter, squamous cell carcinomas constitute the majority, whereas adenocarcinomas are almost exceptions. To the best of our knowledge, only two cases have been reported.

**Case presentation:**

We report the case of a 59-year-old Greek man who presented with persistent cough and chest pain that had started one month prior to a medical appointment. A physical examination, complete laboratory work-up, computed tomography scanning (of the chest, brain, and abdomen), pelvic magnetic resonance imaging, penile ultrasonography, bone scanning, and histological analyses were conducted. Afterward, a lung adenocarcinoma metastatic to the bones, brain, adrenals, lymph nodes, and penis was diagnosed. The primary lesion was a mass of 4cm in diameter in the apical segment of the lower lobe of the right lung. The patient was treated with bone and brain radiotherapy and various cycles of first- and second-line chemotherapy, and partial response was achieved five months after the initial appointment.

**Conclusions:**

Although these metastatic sites are well known to occur from a primary pulmonary malignancy, penile metastasis is extremely rare. Its identification requires prompt awareness by the physician despite the dismal prognosis. Furthermore, since the penis usually is omitted from the physical examination and lung cancer is the leading cause of cancer-related deaths, more penile metastases may be detected in the future, making early detection and appropriate management of great importance.

## Introduction

Metastatic neoplasms of the penis are extremely rare. To the best of our knowledge, about 300 have been reported worldwide. They are associated with advanced disease and poor prognosis, regardless of the tumor origin. When metastatic neoplasms do occur, the primary sites are the urinary bladder (in 30% to 35% of cases), the prostate (in 28% to 30%), the rectosigmoid colon (in 13%), the kidneys (in 8% to 10%), and the testes (in 5%) [1]. In the remaining cases, the primary tumor is in the gastrointestinal or respiratory tract. When the latter is involved, lung cancer is the leading cause, and among the various histotypes, the vast majority are due to squamous cell carcinoma (SCC) [2]. In this paper, we report a rarer case of lung adenocarcinoma that spread to the penis. Also, we perform a review of all similar cases that have been reported in the literature.

In a search of the MEDLINE database for reports of lung cancer that metastasized to the penis, we retrieved 24 cases [2,3], the vast majority being SCCs. Among these cases were two cases of adenocarcinoma, one of epidermoid carcinoma, one of large cell carcinoma, one of epithelial carcinoma, and one of small cell carcinoma. The reason that SCC is the primary histological pattern in most cases is unclear, but the fact that adenocarcinoma is a rare occurrence probably relates to its predominance in women. The first reported case of penile metastasis independent of origin was published by Eberth [4] in 1870.

## Case presentation

A 59-year-old Greek man was admitted to our hospital nine months prior to the submission of this case report. He was complaining of persistent cough and chest pain that had started one month prior to a medical appointment. A physical examination on admission revealed enlarged left supraclavicular lymph nodes (approximately 3cm) and an asymptomatic firm subcutaneous mass on the dorsum of his penis, close to the glans, just left of the midline. No pain on his penis was mentioned at that time. Laboratory findings showed elevated serum levels of carcinoembryonic antigen (37.3ng/ml), neuron specific enolase (35.2ng/ml), and lactate dehydrogenase (1.590IU/L) and slightly elevated levels of creatinine (1.8mg/dL).

**Figure 1:**
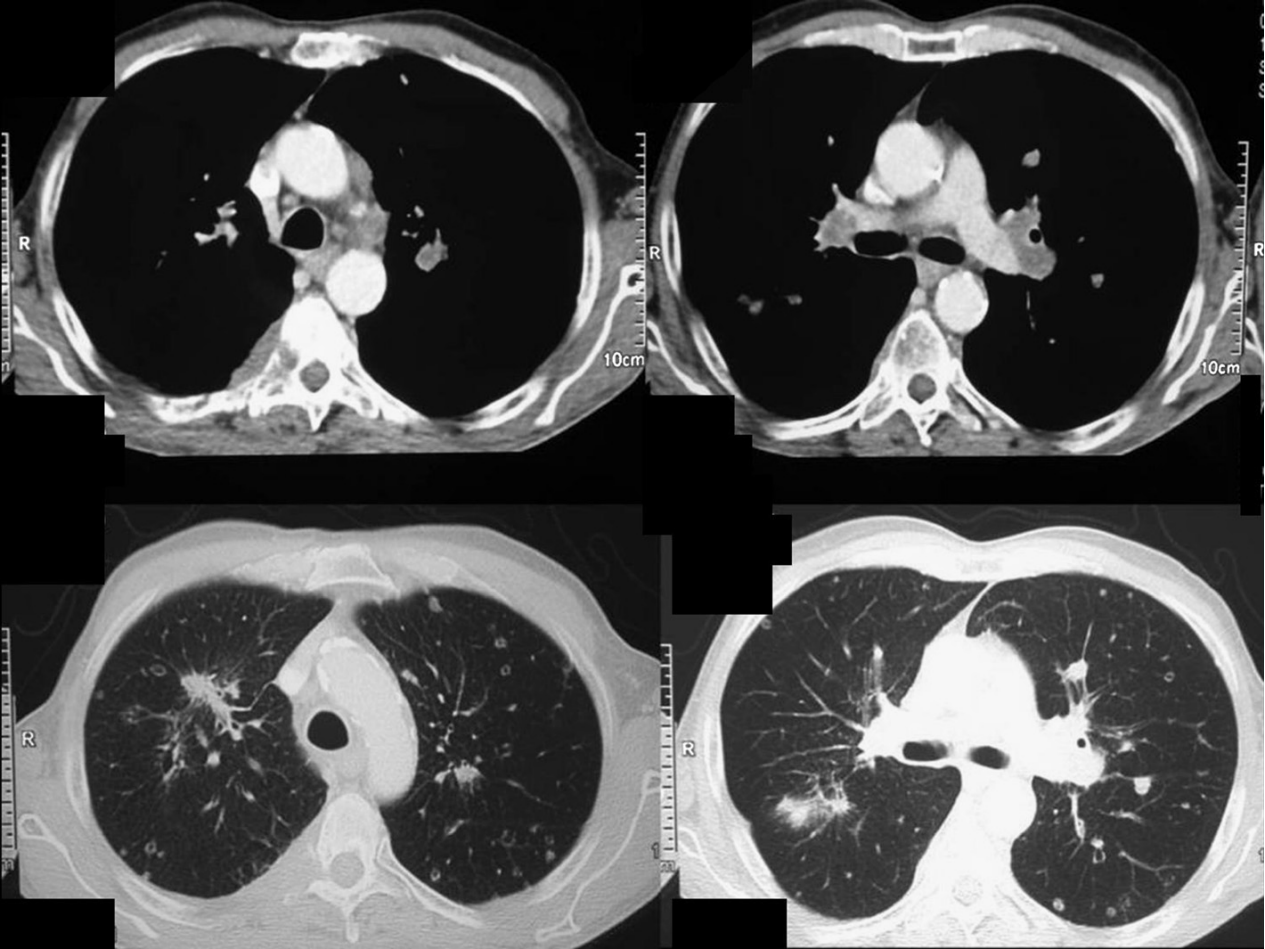
**Computed tomography images of the thorax.** (**a**) Enlarged lymph nodes in the aortopulmonary window. (**b**) Enlarged hilar nodes. (**c**) Spiculated mass in the apical segment of the right upper lobe and metastatic foci. (**d**) Metastatic pulmonary nodules.

A chest plain radiograph and chest computed tomography (CT) demonstrated a spiculated lung mass (with a maximum diameter of 4cm) on the apical segment of the lower right lobe and multiple metastatic pulmonary nodules, consistent with carcinoma of the lung, as well as enlarged mediastinal lymph nodes (with an aortopulmonary window of 1.5cm and bilateral hilar nodes with a maximum diameter of 2.1cm) and left supraclavicular nodes (of approximately 1cm) (Figure 1). A CT scan of the brain revealed no abnormalities, whereas a CT scan of the abdomen showed bilateral adrenal masses, which were larger on the left and were consistent with metastatic disease from lung carcinoma, and enlargements of para-aortic, peripancreatic, and liver portal lymph nodes. The bone scanning and the CT scans of the chest and abdomen revealed osteolytic lesions at the right side of the bodies of T5-T6 thoracic vertebrae and in the posterolateral arches of sixth, seventh, eighth, and ninth ribs, a mass invading the spinal cord, and another mass (with a maximum diameter of 4cm) located next to the ninth right rib.

**Figure 2:**
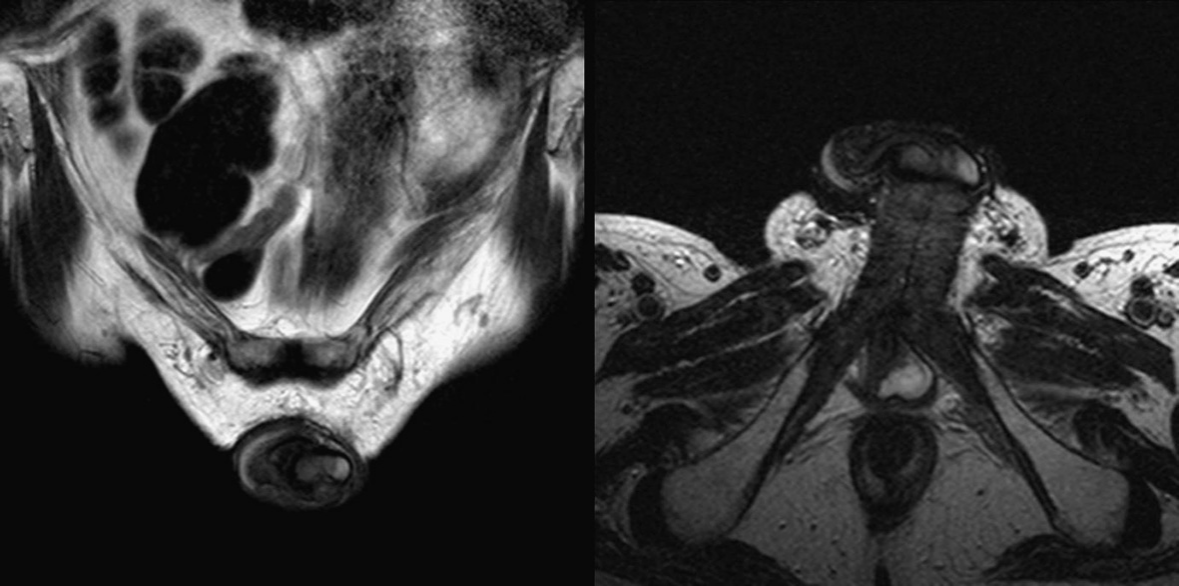
**Magnetic resonance images of the pelvis.** In this high-intensity area on T2, a marginal obscuration of the Buck’s fascia appears thickened around the left corpus cavernosum. (**a**) Coronal image. (**b**) Transverse image.

Magnetic resonance images of the pelvis (Figure 2) demonstrated a high-intensity area on T2 and a marginal obscuration of the Buck’s fascia (with dimensions of 2.5×4.5mm), which appears thickened around the left corpus cavernosum. This finding was confirmed by ultrasound imaging of the penis. The patient subsequently underwent a CT-guided percutaneous puncture of the mass in the left adrenal and para-aortic lymph node. A cytological examination confirmed metastatic adenocarcinoma of the lung (strong immunoreactivity against thyroid transcription factor 1, or TTF-1).

**Figure 3:**
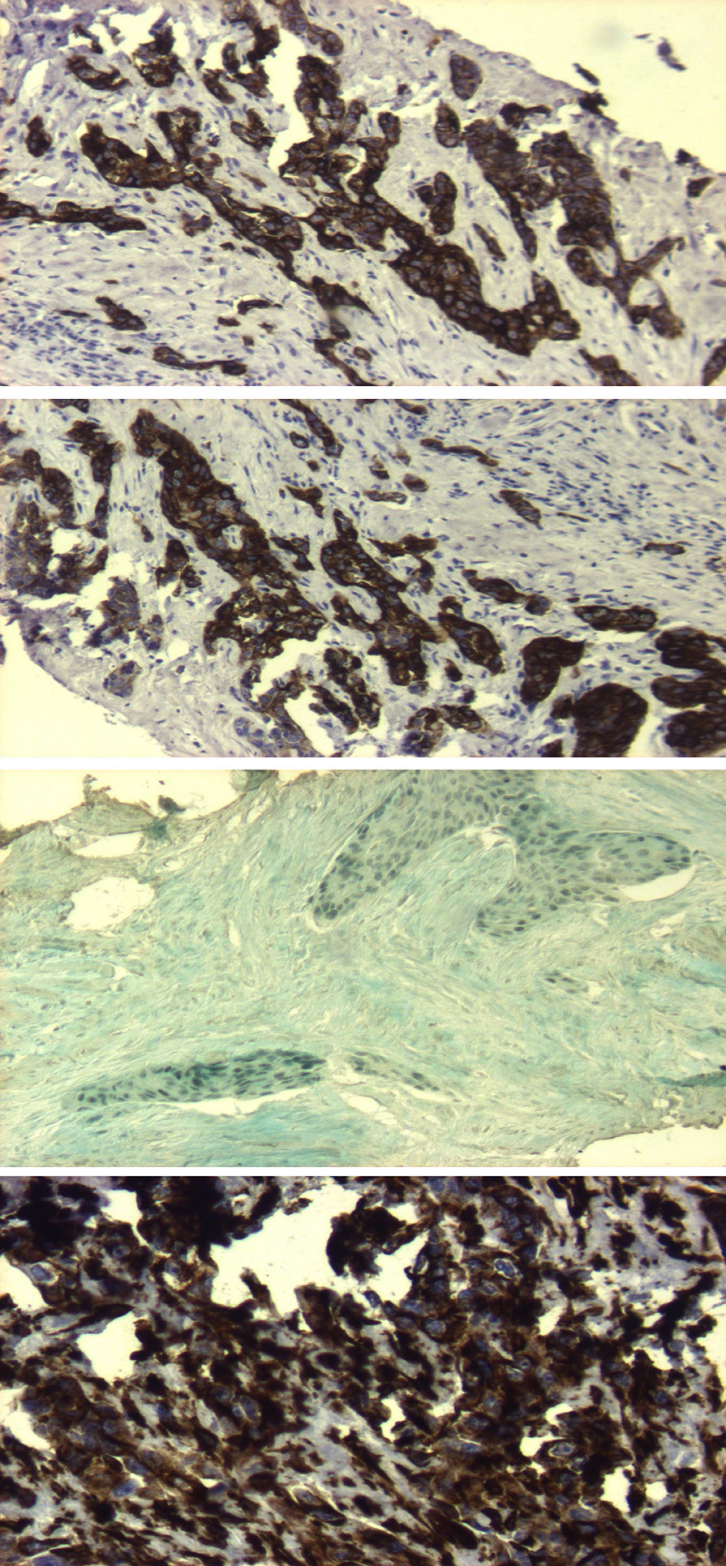
Histopathology specimens of the lesion in the left corpus cavernosum (CK7, CK8, thyroid transcription factor 1, and vimentin).

A penile malignancy was suspected, and a biopsy of the corpus cavernosum penis was performed. The biopsy led to a histological diagnosis of adenocarcinoma (Figure 3). The histology of the specimen was consistent with that of previous lung cancer. Two longitudinal tissue samples from the corpus spongiosum – which measured 1 and 1.8cm, respectively – were obtained for examination. Histology revealed connective tissue with invasion by clusters of cells with marked cellular atypia. The cells showed strong and diffuse immunoreactivity against CK7, CK8, vimentin, and CK34βE12 and focal positivity against TTF-1 and no expression of CK5/6 and CK20 antibodies. The morphologic features and the immunophenotype of the neoplastic cells indicate a poorly differentiated pulmonary carcinoma metastatic to the penile corpus spongiosum (Figure 3). Therefore, the patient was considered to have penile metastasis from lung adenocarcinoma.

Radiotherapy was used to treat the metastatic bone lesions three months after the medical appointment. Three weeks later, the patient was referred to us for treatment of lung cancer. He was treated with chemotherapy consisting of carboplatin (area under the curve of 5) and pemetrexed (500mg/m^2^) given on day 1 in a cycle of three weeks (q21 days). After the third cycle of chemotherapy, restaging of the disease was performed as the clinical performance status was worsening; a chest CT scan confirmed progressive disease with several new lung lesions, but the lymph nodes of the mediastinum as well as the abdominal ones were minimized. Additionally, a CT scan of the brain showed metastatic lesions at the left frontal lobe and the corona radiata. After completion of the total brain irradiation, second-line chemotherapy was administered with paclitaxel (90mg/m^2^) given weekly on days 1, 8, and 15 (q28 days) for two more cycles. Restaging showed partial response (lung lesions, penile lesions, and left supraclavicular lymph nodes), but bone marrow depression followed.

## Discussion

In patients with recurrent or disseminated cancer, metastases simulating primary penile cancer are still unusual, although the penis is a highly vascularized organ that sometimes may harbor subclinical metastatic foci. Abeshouse and Abeshouse [5], in 1961, concluded that, owing to the rarity of metastatic spread from osseous, sarcomatous, and lymphosarcomatous primary malignant tumors, direct arterial dissemination is seldom responsible for secondary tumors to the penis [6]. Several mechanisms by which a tumor can secondarily affect the penis have been described; apart from the retrograde lymphatic route of spread, venous retrograde spread is the commonest route, owing to the generous communication between the pelvic venous plexuses and the penile dorsal venous system [7-9]. Direct tumor extension also has to be considered a possible mechanism of tumor involvement, especially in large and advanced carcinomas. Neoplasms secondarily invading the penis by direct anatomical extension would be detected at the penile root or base of the shaft. However, most of the metastatic tumors are located in the middle to distal portion of the shaft, suggesting that direct extension is not a frequent mechanism of penile metastasis. Arterial spread is another possible route of spread. In practice, iatrogenic spread after certain medical-therapeutic interventions of the urinary tract is unlikely to explain the majority of the cases reported [7,9]. Differentiating primary carcinoma of the penis from a secondary one is of crucial importance since treatment procedures for primary lesions and prognosis are not the same as those of metastatic disease.

Metastatic tumors to the penis give rise to various clinical signs and symptoms, such as severe penile or perineal pain or both (7% to 10%), swelling of the penis, erectile dysfunction, priapism (up to 45%), enlargement of inguinal lymph nodes, tenderness, ulcer formation, mass (usually rigid, smooth, immobile, and painless), poor or weak urinary stream, urinary infections, dysuria, pollakisuria, hematuria (less than 10%), urinary retention, or other unspecific obstruction symptoms. Penile mass is a common sign of penile tumor, although priapism is usually the most common symptom, resulting in thrombosis or emboli or congestion of the circulation (‘malignant priapism’). In contrast, perineal pain is not a typical symptom. Ulcer formation is usually a sign of disseminated disease and dismal prognosis. Most of the time, the quality of life is extremely impaired and the patient seeks help and palliation, although metastasis to the penis usually is considered a late complication and often is associated with multiple metastases in many distant organs.

Diagnosis of metastasis to the penis must be established on biopsy. To differentiate metastatic lesions from primary penile tumors, an adequate specimen is needed as early as possible. A penile enlargement or mass does not always mean malignancy. Any of the following could be on the list of differential diagnosis of the clinician: benign tumors, syphilitic chancre, venereal or other infectious ulcerations, idiopathic priapism, Peyronie’s plaque, candidiasis, cavernositis, tuberculosis of the penis, sclerosing lipogranuloma, and other specific and non-specific inflammation together with all of the hematologic, metabolic, and pharmacological causes of priapism [2]. It is important, though, to differentiate primary penile carcinoma from secondary metastatic disease since primary tumors are curable in a high percentage of cases, whereas the majority of patients with metastatic lesions die of their primary cancer within weeks or months or a year, regardless of the type of therapy.

Several diagnostic modalities can be used to confirm the clinician suspicion. Magnetic resonance imaging yields higher accuracy in the differential diagnosis in comparison with ultrasound or CT scans and should be preferred. Other imaging techniques, such as cavernosonography, are not used nowadays, mainly because they do not provide higher diagnostic accuracy in comparison with other non-invasive procedures and are associated with a considerable complication rate [10].

Therapeutic modalities that aim to provide local and systemic control of the disease are surgery, radiotherapy, and chemotherapy alone or in combination. All treatments may be considered palliative as published clinical cases report improvement of patient symptoms without any important prolongation in survival. Polonged survival is often possible only when a distal penile lesion or nodule is resectable; limited surgical excision or radiotherapy is usually the most useful modality. Partial or total penectomy will be required with highly invasive neoplasms involving the corporal bodies. Penile ulcer has to be taken out by local excision. However, surgical treatment is also recommended as an option after other treatments have failed and as a cure for intractable pain. A penile dorsal nerve block using local anesthesia may be of some benefit to control pain. Suprapubic urinary diversion would be the intended treatment for those patients with dysuria and urinary retention. Radiotherapy is used to reduce the size of the lesion as well as to improve pain control [2-10]. Second-line chemotherapy with docetaxel, paclitaxel, vinorelbine, gemcitabine, irinotecan, and gefitinib has an unknown curative effect. In light of a dismal prognosis, these patients should be treated mainly with palliative therapy to relieve the intolerable symptoms. Anxiety and pain require parenteral opioids and anxiolytics.

## Conclusions

Tumors metastatic to the penis are rare. Common primary sites are the genitourinary tract (mainly, urinary bladder) and the lower gastrointestinal tract (especially, rectum and sigmoid colon). Metastasis to the penis is extremely rare in squamous and non-squamous lung cancer. The fact that the penis often is not examined routinely leads to the low incidence of the disease. When recognized, penile metastatic lesions, regardless of the tumor origin, have traditionally been associated with advanced, metastatic, and disseminated disease and ominous prognosis. Palliative care and relief of symptoms must be the priority of the clinician since no treatment modality – surgery, radiotherapy, or chemotherapy alone or in combination – has been shown to significantly improve the overall survival. For patients with penile metastasis, the cancer-related death rate is still high. Since lung cancer remains the leading cause of cancer-related deaths worldwide and more penile metastases will be detected in the future, early detection and appropriate management of penile metastasis will be of great importance.

## Consent

Written informed consent was obtained from the patient for publication of this manuscript and accompanying images. A copy of the written consent is available for review by the Editor-in-Chief of this journal.

## Abbreviations

CT, Computed tomography; SCC, Squamous cell carcinoma; TTF-1, Thyroid transcription factor 1.

## Competing interests

The authors declare that they have no competing interests.

## Authors’ contributions

CK was the main author of the paper and the referring radiologist. NP, EM, and CSB, radiologists, contributed to the manuscript by revising it and adding important information. DA and SL are assistant radiologists who contributed to analysis of manuscript data. NK is head of the histopathology department in our hospital. SX is head of the oncology department in our hospital. All authors read and approved the final manuscript.

## References

[B1] Berger AP Rogatsch H Hoeltl L Steiner H Bartsch G Hobish A Late penile metastasis from primary bladder carcinoma Urology 2003 62 145xix 145xxi 10.1016/s0090-4295(03)00239-5 12837452

[B2] Zheng FF Zhang ZY Dai YP Liang YY Deng CH Tao Y Metastasis to the penis in a patient with adenocarcinoma of lung, case report and literature review Med Oncol 2009 26 228 232 10.1007/s12032-008-9113-8 18975150

[B3] Haliloglu AH Haliloglu N Akpinar EE Ataoglu O Erectile dysfunction: initial symptom of a patient with lung cancer J Sex Med 2011 8 3511 3514 10.1111/j.1743-6109.2009.01431.x 19674251

[B4] Eberth CJ Krebsmetastasen des corpus cavernosum penis. (Cancer metastases of the corpus cavernosum of penis) Virchows Arch 1870 51 145

[B5] Abeshouse BS Abeshouse GA Metastatic tumors of the penis: a review of the literature and a report of two cases J Urol 1961 86 99 100 13681031 10.1016/S0022-5347(17)65117-6

[B6] Bonaminio A Shingleton WB Squamous cell carcinoma of the lung with metastasis to the penis South Med J 1995 88 761 762 10.1097/00007611-199507000-00014 7597484

[B7] Chaux A Amin M Cubilla AL Young RH Metastatic tumors to the penis: a report of 17 cases and review of the literature Int J Surg Pathol 2011 19 597 606 10.1177/1066896909350468 20075023

[B8] Cherian J Rajan S Thwaini A Elmasry Y Shah T Puri R Secondary penile tumours revisited Int Semin Surg Oncol 2006 3 33 10.1186/1477-7800-3-33 17032461 PMC1618838

[B9] Paquin AJ Roland SI Secondary carcinoma of the penis: a review of the literature and a report of nine new cases Cancer 1956 9 626 632 10.1002/1097-0142(195605/06)9:3<626::AID-CNCR2820090330>3.0.CO;2-C 13330017

[B10] Lau TN Wakeley CJ Goddard P Magnetic resonance imaging of penile metastases: a report on five cases Australas Radiol 1999 43 378 381 10.1046/j.1440-1673.1999.433690.x 10901942

